# Male urethral sarcoma: a case report and literature review

**DOI:** 10.1590/S1679-45082015RC2992

**Published:** 2016

**Authors:** Magno Almeida Nogueira, Guilherme Campelo Lopes dos Santos, Roberto Iglesias Lopes, Octavio Henrique Arcos Campos, Marcos Francisco Dall’Oglio, Alexandre Crippa Sant’Anna

**Affiliations:** 1 Hospital do Servidor Público Municipal de São Paulo, São Paulo, SP, Brazil.; 2 Hospital das Clínicas, Faculdade de Medicina, Universidade de São Paulo, São Paulo, SP, Brazil.

**Keywords:** Sarcoma, Urethral neoplasms, Hematuria, Case reports

## Abstract

Urethral tumors are rare and aggressive. They usually affect men (2:1) and occur more commonly in white (85% of cases). Soft tissue sarcomas are a heterogeneous group of tumors that arise from embryonic mesoderm. It represents 1% of all cases of urinary tract malignancies and rarely primary affect the ureter. We report a case of male urethral sarcoma. To date, only two similar cases have been published in literature.

## INTRODUCTION

Urethral tumors are rare and aggressive, they occur more frequently in men (2:1) and often affect whites (85% of cases).^1,2^

Approximately 600 cases of urethral cancer in men are reported in the world literature. The epidermoid carcinoma is the most common histological type (80% of cases), followed by transitional cell cancer (15%) and adenocarcinomas (5%). The most affected local is bulbomembranous urethra followed by penile and prostatic urethra.^2^ Urethral cancer is often diagnosed between the age of 50-79 years.^2,3^

The disease has an unspecific presentation and, according to some case series reported, the most common symptoms are hematuria, dysuria, urinary retention and urinary incontinence.^4^

Soft tissue sarcomas constitute a heterogenous group of tumors that appear in the embryonic mesoderm and account for 1 to 2% of all malignant disease of the urinary tract.^5^

In general sarcomas most common forms are liposarcoma, malignant histiocytoma and leiomyosarcoma.^6^ The most frequent types of genitourinary sarcomas are leiomyosarcoma, liposarcoma and rhabdomyosarcoma.^6^

We report a case of sarcoma of the male urethra that, so far, only two similar cases have been reported in the literature.^7,8^

## CASE REPORT

We report a case of 73-year-old white man with history of myocardial infarction (MI), which occurred 3 years earlier, and who was diagnosed with systemic arterial hypertension. He was not a smoker and did not consume alcohol. The patient’s complain was macroscopic hematuria that started intermittent and then continuing that lasted for 1 year.

**Figure 1:**
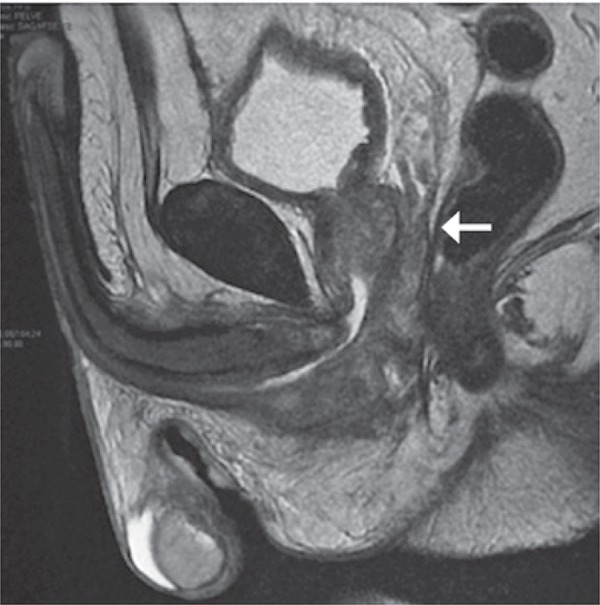
Nuclear magnetic ressonance of the pelvis showing the urethral tumor (arrow)

At physical examination no visceromegaly or lymphadenomegaly was observed. The urological exam showed urethrorrhagia without sings of macroscopic lesions. Rectal examination revealed a 30g prostate without nodularity and painless. Patient had normal blood count and coagulogram, early-onset chronic renal failure (urea was 49mg/dL and creatinine was 1.5mg/dL). Total abdominal ultrasound was normal and pelvis examination with contrast showed an extensive injury in posterior urethra that invaded the prostate, bladder and perivesical tissue (Figure 1), but no changes were seen in the upper urinary tract or distant metastasis. The cystoscopy showed a brown injury in the posterior urethra (membranous and prostatic). A biopsy was performed and the result revealed a mesenchymal neoplasia with myxoid degeneration area and high mitotic index.

**Figure 2:**
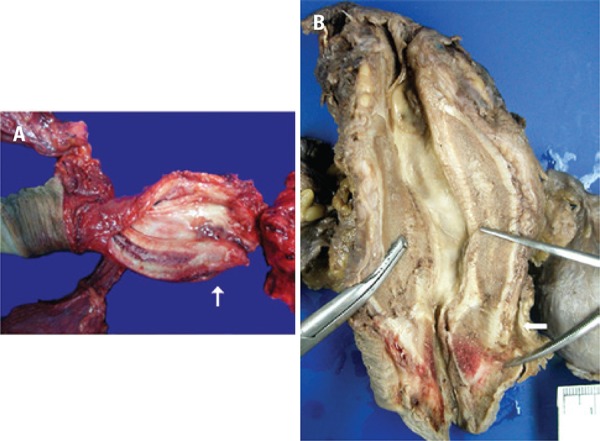
Posterior urethral sarcoma (indicated with arrows). Thickening and hardening of the affected area

The patient underwent a cystoprostatectomy with removal of the testicles, pelvic lymphadenectomy and ureteroileostomy using the Bricker method (Figure 2). After the procedure the patient had a good progress, recovery of physiological functions and adequate diuresis. However, on the 10th postoperative day he died after a new episode of MI.

**Figure 3:**
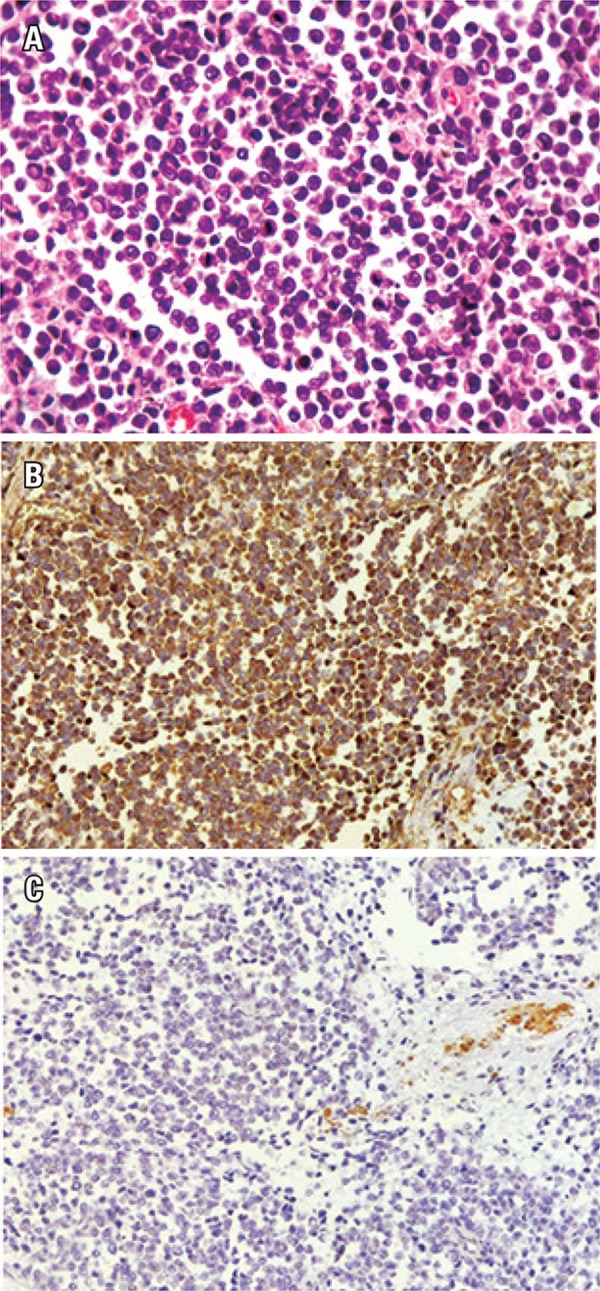
(A) High degree urethral sarcoma; (B) Immunohistochemical analysis that revealed strong positivity of vimentin; (C) Urethral tumor also showing positivity for desmin

The anatomopathological study showed a high histological degree pleomorphic sarcoma, measuring approximately 20.0cm, in the bulbar urethra with neoplasia affecting the bladder base, periprostatic region, seminal vesicles, base of the penis and cavernous bodies (Figure 3). The urethral tumor had high cellularity, high mitotic index, 30% necrosis of tumor volume and intense anaplastic effect. The perivescial surgical margins and the right ureter was compromised. The left urethral margin was free of malignancy. No metastasis was observed in 15 dissected lymph nodes in the pelvic lymphadenectomy. Mesenchymal cells vimentin and desmin were positive in the immunohistochemical analysis, but other markers such as cytokeratin, S-100, CD 117, CD 30, CD 20, CD 3 and CD 56 were negative. This finding excluded other origins such as epithelial, hematological and neural tumors.

## DISCUSSION

Survival rates of patients with genitourinary sarcomas is worse compared with other soft tissue regions.^9^ Prognosis is relatively poor and can be explained by the high proportion seen in high degree tumors, large proportion of patients with metastatic disease, large tumor and the area affected. In addition, the rarity and heterogeneity of genitourinary sarcomas can explain the great variability in clinical progress in different subgroups.^10^

Dissemination of urethral cancer follows the anatomic subdivision. The anterior urethra has a lymphatic drainage system for superficial and deep inguinal region. Posterior urethra drains the lymphatic ganglion of external iliac artery, hypo gastric, and internal obturator muscle.^10^

Late diagnosis is seen in one third of patients with inguinal lymphatic ganglion metastasis and in 20% of those with pelvic ganglion metastasis. The most common distant metastasis appears in lungs, liver and bones.^10^

Anterior tumors present better prognosis reaching up to 60% in 5 years. Tumors <2cm have 81% of survival for 5 years. On the other hand, tumors of posterior urethra have 10% survival rate for 5 years if <2cm, 37% of survival for 5 years when presenting tumors 2 to 4cm and only 7% when >4cm. In general, in a stage higher than T2 the survival rate for 5 years is observed in <26%.^10^

Histological characteristics of genito-urinary sarcomas are quite variable. Most of them present high degree of differentiation and isolated focus of squamous metaplasia.^10^

To date, only two cases of sarcoma of the male urethra have been described in the literature. The first cases was a 78-year-old man with ulcerative injury in urethra.^7^ The second was a 65-year-old man who underwent a perineal uretrostomy to treat a distal urethral stenosis ^8^(Chart 1).

**Chart 1:** Case reported in the literature about sarcoma of the male urethra

Patient	Age (years)	Local	Size	Treatment	Progress
1^o^ patient^(7)^	78	Posterior urethra	Not evaluated	Vesical drainage	Not evaluated
2^o^ patient^(8)^	65	Bulbar urethra	6cm	Pelvic exenteration, chemotherapy and adjuvant radiotherapy	Death after 7 months

The main treatment for urethral cancer is surgical excision. Anterior urethral cancer has a better surgical control and prognosis than the posterior urethral that often present association with local invasion and distant extensive metastasis.^2^

More conservative procedures can be acceptable in selected cases with superficial injuries, papillary tumors or low degree tumors.^2^ In case of infiltrating tumors of sponge’s body located in distal half of the penis, the partial penectomy can be a treatment option.^2^ If the disease is invasive, extending for more than half of the penile urethra, the radiotherapy can be a treatment option for unresectable lesions.^2^

Radiotherapy and chemotherapy can be applied to tumors located in bulbocavernosus urethra even for those occurring in the prostatic urethra. In cases of advanced injuries, the treatment applied is extensive surgery with chemotherapy and adjuvant radiotherapy. The isolated chemotherapy is the only option for cases of extensive metastasis.^2^

In our case the diagnosis was advanced urethral sarcoma (T4N0M0), with positive surgical margin in a clinically severe patient with cardiomyopathy that led to an unfavorable outcome. The MI presented by the patient in early postoperative period did not enable the adjuvant therapy with radiotherapy and chemotherapy. It becomes clear that these tumors are extremely aggressive and have poor prognosis.

## References

[1] Swartz MA, Porter MP, Lin DW, Weiss NS (2006). Incidence of primary urethral carcinoma in the United States. Urology.

[2] Walsh PC, Retik AB, McDougal WC, Wein AJ, Kavoussi LR, Novick AC, Partin AW, Peters CA (2012). Surgery of penile and urethral carcinoma. Campbell´s Urology.

[3] Stojadinovic A, Leung DH, Allen P, Lewis JJ, Jaques DP, Brennan MF (2002). Primary adult soft tissue sarcoma: time-dependent influence of prognostic variables. J Clin Oncol.

[4] Touijer AK, Dalbagni G (2004). Role of voided urine cytology in diagnosing primary urethral carcinoma. Urology.

[5] Srinivas V, Sogani PC, Hajdu SI, Whitmore WF (1984). Sarcomas of the kidney. J Urol.

[6] Sexton WJ, Lance RE, Reyes AO, Pisters PW, Tu SM, Pisters LL (2001). Adult prostate sarcoma: the M. D. Anderson Cancer Center Experience. J Urol.

[7] Mark EG (1992). Primary sarcoma of the male urethra: report of a case. Ann Surg.

[8] Ahallal Y, Tazi MF, Khallouk A, Tazi E, Benlemlih A, El Fassi MJ (2009). Primary leiomyosarcoma of the male urethra: a case report. Cases J.

[9] Clark MA, Fisher C, Judson I, Thomas JM (2005). Soft-tissue sarcomas in adults. N Engl J Med.

[10] Dotan ZA, Tal R, Golijanin D, Snyder ME, Antonescu C, Brennan MF (2006). Adult genitourinary sarcoma: the 25-year Memorial Sloan-Kettering experience. J Urol.

