# Supplementary material for: Male urethral sarcoma: a case report and literature review
Source: Einstein (Sao Paulo). 2016 Jan-Mar;14(1):64–6. doi: 10.1590/S1679-45082015RC2992 (PMC4872920; doi:10.1590/S1679-45082015RC2992)
Supplement: Translation

# Translation: Sarcoma de uretra masculina: relato de caso e revisão de literatura

**Authors:** Magno Almeida Nogueira, Guilherme Campelo Lopes dos Santos, Roberto Iglesias Lopes, Octavio Henrique Arcos Campos, Marcos Francisco Dall’Oglio, Alexandre Crippa Sant’Anna

**Affiliations:** 1 Hospital do Servidor Público Municipal de São Paulo, São Paulo, SP, Brasil.; 2 Hospital das Clínicas, Faculdade de Medicina, Universidade de São Paulo, São Paulo, SP, Brasil.

**Keywords:** Sarcoma, Neoplasias uretrais, Hematúria, Relatos de casos

## Abstract

Os tumores de uretra são muito raros e bastante agressivos. Acometem mais frequentemente homens (2:1) e são mais comuns na raça branca (85% dos casos). Os sarcomas de tecidos moles são um grupo heterogêneo de tumores que surgem a partir da mesoderme embrionária e representam 1% de todos os casos de doença maligna urinária, raramente acometendo a uretra primariamente. Relamos aqui um caso extremamente raro de sarcoma uretral masculino com somente dois semelhantes publicados na literatura.

## INTRODUÇÃO

Os tumores de uretra são muito raros e bastante agressivos. Acometem mais frequentemente homens (2:1) e são mais comuns na raça branca (85% dos casos).^1,2^

São relatados aproximadamente 600 casos de câncer uretral masculino na literatura mundial. O carcinoma epidermoide é o tipo histológico mais comum (80% dos casos), seguido do carcinoma de células transicionais (15%) e dos adenocarcinomas (5%). O local mais acometido é a uretra bulbomembranosa, seguida da uretra peniana e prostática.^2^ A faixa etária do diagnóstico de tumor uretral maligno é de 50 a 79 anos.^2,3^

A doença tem apresentação inicial inespecífica e, de acordo com algumas séries de casos relatados, os sintomas mais comuns são hematúria, disúria, retenção urinária e incontinência urinária.^4^

Os sarcomas de tecidos moles são um grupo heterogêneo de tumores que surgem a partir da mesoderme embrionária, que representa 1 a 2% de todos os casos de doença maligna urinária.^5^

Os sarcomas, em geral, possuem como formas mais comum o lipossarcoma, seguido do histiocitoma maligno e do leiomiossarcoma.^6^ Em relação aos sarcomas geniturinários, o tipo mais frequente é o leiomiossarcoma, seguido do lipossarcoma e do rabdomiossarcoma.^6^

Relatamos e discutimos aqui um caso de sarcoma de uretra masculino, que, por sua raridade, tem apenas dois casos relatados.^7,8^

## RELATO DE CASO

Paciente de 73 anos, do sexo masculino, raça branca, com história de infarto agudo do miocárdico (IAM) há 3 anos e diagnóstico de hipertensão arterial sistêmica, sem história de tabagismo e etilismo. Apresentou-se com queixa de hematúria macroscópica, inicialmente intermitente e, posteriormente, contínua, de início há 1 ano.

No exame físico, não foram notadas visceromegalias ou linfonodomegalias. Ao exame urológico, notou-se uretrorragia, sem sinais de lesões macroscópicas. O toque retal revelou próstata de 30g sem nódulos, indolor. O paciente apresentava hemograma e coagulograma normais, função renal com doença renal crônica incipiente (ureia 49mg/dL e creatinina 1,5mg/dL). O ultrassom de abdome total era normal, porém a ressonância nuclear magnética de abdome e pelve com contraste evidenciou lesão extensa em uretra posterior, que invadia próstata, bexiga e tecido perivesical (Figura 1), sem alteração de trato urinário alto ou metástases a distância. A uretrocistoscopia demonstrou lesão acastanhada na uretra posterior (membranosa e prostática), que, submetida à biópsia, demonstrou neoplasia de padrão mesenquimal, com áreas de degeneração mixoide e alto índice mitótico.

**Figura 1:** Ressonância nuclear magnética de pelve demonstrando tumor acometendo uretra (seta)

O paciente foi submetido à cistoprostatectomia com emasculação, linfadenectomia pélvica e ureteroileostomia à Bricker (Figura 2). Apresentava boa evolução com recuperação das funções fisiológicas e diurese adequada; todavia, apresentou novo episódio de IAM no 10^o^ dia pós-operatório evoluindo a óbito.

**Figura 2:** Sarcoma da uretra posterior (indicado pelas setas). Clinicamente, notavam-se espessamento e endurecimento da região acometida

O estudo anatomopatológico evidenciou sarcoma pleomórfico de uretra bulbar de alto grau histológico, com neoplasia acometendo base de bexiga, região periprostática, vesículas seminais, base de pênis e corpos cavernosos, com maior diâmetro aproximado de 20,0cm (Figura 3). O tumor uretral apresentou alta celularidade, alto índice mitótico, necrose em 30% do volume tumoral e intensa anaplasia. Margens cirúrgicas perivesical e ureteral direita comprometidas. Margem ureteral esquerda livre. Ausência de metástases para 15 linfonodos dissecados na linfadenectomia ilíaca. Na imuno-histoquímica, as pesquisas para os marcadores mesenquimais desmina e vimentina vieram positivos, enquanto pesquisas de outros marcadores, como citoqueratina, S-100, CD 117, CD 30, CD 20, CD 3 e CD 56, foram negativos, excluindo outras origens como epitelial, hematológica e tumores neurais.

**Figura 3:** (A) Sarcoma uretral de alto grau; (B) Imuno-histoquímica que revelou forte positividade de vimentina; (C) O tumor uretral também apresentava positividade para desmina

## DISCUSSÃO

A taxa de sobrevivência de doentes com sarcoma de geniturinário é pior em relação àqueles com sarcoma de tecidos moles em outras localizações.^9^ O prognóstico relativamente pobre pode ser explicado pela maior proporção de tumores de grau elevado, a maior proporção de pacientes que se apresentam com doença metastática, um maior tamanho tumoral e o local anatômico. Além disso, a raridade e a heterogeneidade dos sarcomas geniturinários podem significar grandes diferenças de evolução clínica nos vários subgrupos.^10^

As vias de disseminação do carcinoma uretral seguem sua subdivisão anatômica. A uretra anterior possui drenagem linfática para região inguinal superficial e profunda. A uretra posterior drena para os gânglios linfáticos da artéria ilíaca externa e hipogástrica, e do músculo obturador interno.^10^

Em um terço dos pacientes, o diagnóstico é tardio, com metástases para gânglios linfáticos inguinais, e em 20% para os gânglios pélvicos. Os locais de metástases a distância mais comum são pulmão, fígado e ossos.^10^

Os tumores anteriores são de melhor prognóstico, chegando a 60% em 5 anos. Os tumores <2cm têm sobrevida de 81% em 5 anos. Em contrapartida, tumores da uretra posterior possuem taxa de 10% de sobrevida em 5 anos se <2cm, 37% de sobrevida em 5 anos quando de 2 a 4cm de tamanho e apenas 7% quando >4cm. Acima do estádio T2, costumam apresentar uma taxa de sobrevida <26% em 5 anos.^10^

As características histológicas dos sarcomas urinários são bastante variadas. A maioria contém alto grau de diferenciação com focos isolados de metaplasia escamosa.^10^

Em relação aos sarcomas de uretra masculina, somente dois casos foram descritos na literatura. O primeiro caso é de um homem de 78 anos com uma lesão ulcerada na uretra.^7^O segundo foi de um homem de 65 anos com uretrostomia perineal para tratamento de estenose uretral distal^8^(Quadro 1).

**Quadro 1:** Casos relatados na literatura de sarcoma de uretra no sexo masculino

Paciente	Idade (anos)	Localização	Tamanho	Tratamento	Evolução
1^o^ paciente^(7)^	78	Uretra posterior	Não avaliado	Drenagem vesical	Não avaliado
2^o^ paciente^(8)^	65	Uretra bulbar	6cm	Exanteração pélvica, quimioterapia e radioterapia adjuvante	Óbito após 7 meses

A principal forma de tratamento é a excisão cirúrgica. O carcinoma de uretra anterior possui melhor controle cirúrgico e melhor prognóstico em relação ao da uretra posterior, que apresenta frequente associação de invasão local e extensa metástase a distância.^2^

Procedimentos mais conservadores podem ser aceitáveis em doentes selecionados com lesões superficiais, tumores papilares ou de baixo grau.^2^No caso de tumores infiltrantes do corpo esponjoso localizados em metade distal do pênis, pode-se tentar uma penectomia parcial.^2^Se a doença for invasiva, estendendo-se por mais da metade da uretra peniana, a penectomia total é necessária.^2^

Radioterapia e quimioterapia são opções como tratamento primário ou de forma adjuvante. Em tumores localizados na uretra peniana, a radioterapia entra como tratamento opcional em lesões irressecáveis. Naqueles localizados na uretra bulbo-carvenosa, a radioterapia e a quimioterapia neoadjuvante podem ser realizadas, o mesmo ocorrendo em tumores localizados na uretra prostática. Em casos de lesões avançadas, o tratamento realizado é cirurgia extensa com quimioterapia e radioterapia adjuvante e, nos casos com metástase extensa, quimioterapia isolada é a única opção.^2^

No caso apresentado, o diagnóstico era de sarcoma uretral avançado (T4N0M0), com margem cirúrgica positiva em um paciente com miocardiopatia clinicamente grave, resultando em desfecho desfavorável. O IAM apresentado pelo paciente no período pós-operatório precoce inviabilizou o tratamento adjuvante com radioterapia e quimioterapia. Fica evidente que esses tumores são extremamente agressivos e de mau prognóstico.
